# Preeclampsia Prevalence, Risk Factors, and Pregnancy Outcomes in Sweden and China

**DOI:** 10.1001/jamanetworkopen.2021.8401

**Published:** 2021-05-10

**Authors:** Yingying Yang, Isabelle Le Ray, Jing Zhu, Jun Zhang, Jing Hua, Marie Reilly

**Affiliations:** 1 Department of Women and Children’s Health Care, Shanghai First Maternity and Infant Hospital, Tongji University School of Medicine, Shanghai, China; 2 Department of Medical Epidemiology and Biostatistics, Karolinska Institute, Stockholm, Sweden; 3 Department of Gynecology and Obstetrics, Strasbourg University Hospital, Strasbourg, France; 4 Ministry of Education-Shanghai Key Laboratory of Children’s Environmental Health, Xinhua Hospital, Shanghai Jiao Tong University School of Medicine, Shanghai, China; 5 Department of Obstetrics and Gynecology, Xinhua Hospital, Shanghai Jiao Tong University School of Medicine, Shanghai, China

## Abstract

**Importance:**

Preeclampsia is a leading cause of maternal and perinatal morbidity and mortality worldwide. Within-country studies have reported racial differences in the presentation and outcome, but little is known about differences between countries.

**Objective:**

To compare preeclampsia prevalence, risk factors, and pregnancy outcomes between the Swedish and Chinese populations.

**Design, Setting, and Participants:**

This cross-sectional study compared deliveries from the Swedish national Medical Birth Register (2007-2012) and the China Labor and Delivery Survey (2015-2016). The Swedish Medical Birth Register records maternal, pregnancy, and neonatal information for nearly all deliveries in Sweden. The China Labor and Delivery Survey was conducted throughout China, and these data were reweighted to enable national comparisons. Participants included 555 446 deliveries from Sweden and 79 243 deliveries from China. Data management and analysis was conducted from November 2018 to August 2020 and revised in February to March 2021.

**Exposures:**

Maternal characteristics, parity, multiple gestation, chronic and gestational diabetes, cesarean delivery.

**Main Outcomes and Measures:**

Preeclampsia prevalence and risk factors, overall and for mild and severe forms and rates of adverse neonatal outcomes compared with pregnancies with no gestational hypertension.

**Results:**

The 555 446 Swedish pregnancies and 79 243 Chinese pregnancies had mean (SD) maternal age of 30.9 (5.3) years and 28.6 (4.6) years, respectively. The overall prevalence of preeclampsia was similar in Sweden and China, 16 068 (2.9%) and 1803 (2.3%), respectively, but with 5222 cases (32.5%) considered severe in Sweden and 1228 cases (68.1%) considered severe in China. Obesity (defined as BMI ≥28 in China and BMI ≥30 in Sweden) was a stronger risk factor in China compared with Sweden (China: odds ratio [OR], 5.12; 95% CI, 3.82-6.86; Sweden: OR, 3.49; 95% CI, 3.31-3.67). Nulliparity had a much stronger association with severe preeclampsia in Sweden compared with China (Sweden: OR, 3.91; 95% CI, 3.65-4.18; China: OR, 1.65; 95% CI, 1.20-2.25). The overall stillbirth rate for singleton in China was more than 3-fold higher than in Sweden (846/77 512[1.1%] vs 1753/547 219 [0.3%], *P* < .001), and 10-fold higher among women with preeclampsia (66/1652 [4.6%] vs 60/14 499[0.4%], *P* < .001).

**Conclusions and Relevance:**

In this study, the prevalence rates of preeclampsia in Sweden and China were similar, but women in China had more severe disease and worse pregnancy outcomes than women in Sweden. The associations of obesity and nulliparity with preeclampsia suggest a role for lifestyle and health care factors but may reflect some differences in pathophysiology. These findings have relevance for current efforts to identify high-risk pregnancies and early serum markers because the value of risk prediction models and biomarkers may be population specific.

## Key Points

**Question:**

Does preeclampsia differ in prevalence, risk factors, or pregnancy outcomes in the Swedish and Chinese populations?

**Findings:**

In this cross-sectional study of 634 689 pregnancies, preeclampsia had a similar prevalence in Sweden (2.8%) and China (2.2%), but two-thirds of cases were mild in Sweden, while two-thirds of cases were severe in China, and notable differences were found for the association of nulliparity and body mass index. The stillbirth rate for preeclampsia in China was almost 10-fold higher than in Sweden.

**Meaning:**

This comparison adds to the global literature on preeclampsia, helps to fill the data gap for Asian populations, and suggests an important role for the management of maternal care.

## Teaser

This cross-sectional study compared preeclampsia prevalence, risk factors, and pregnancy outcomes between the Swedish and Chinese populations.

## Introduction

Preeclampsia is a major maternal health issue worldwide that is responsible for maternal and neonatal severe morbidity and mortality and has substantial contributions to prematurity of the fetus and long-term cardiovascular disease (CVD) in the mother.^1^ Although the definition of preeclampsia varies between countries, most are similar to the definition provided by the International Society for the Study of Hypertension in Pregnancy (ISSHP),^2,3^ which is predominantly used worldwide. The ISSHP defines preeclampsia as the presence of new-onset hypertension and proteinuria or other end-organ damage occurring after 20 weeks of gestation,^4^ with eclampsia defined as the development of grand seizures in a woman with preeclampsia. Preeclampsia affects an estimated 4.6% of pregnancies globally.^5^

The etiology of preeclampsia is complex, and a role for maternal and fetal and/or paternal genetic determinants has been suggested by early family-based studies.^6,7^ The association of ethnicity with preeclampsia severity has generally been investigated in within-country studies in multicultural settings, comparing mostly African American, Hispanic, and White subgroups in the United States.^8,9^ In addition to the sparsity of data about Asian or Chinese women as an ethnic subgroup in those studies, there are no population-based studies of preeclampsia among women in China. However, the many racial and ethnic differences that were noted in a recent review^10^ suggest that there could be important differences between women in China and Europe. Ethnic differences reflect many factors, such as lifestyle, socioeconomic status, cultural norms, and the seeking and provision of medical care,^11^ which can have a greater association with differences between countries than racial or genetic factors. However, genetic studies have suggested some association with preeclampsia, including variations in MS-like tyrosine kinase 1 and vascular endothelial growth factor C^12^ and a microsatellite variation in the heme-oxygenase 1promoter in a Finnish cohort^13^ but not in a Chinese cohort.^14^ Thus, based on the various types of evidence available from current research and the potential impact of health care infrastructure on diagnosis, management, and related complications of preeclampsia, we hypothesized that the etiology, severity, and consequences of preeclampsia may differ in a country-level comparison of China and Sweden.

The national registers that enable between-country comparisons of population health are generally available in high-income countries, and it is rare to have country-level data for middle-income countries. A systematic review of global burden of preeclampsia and eclampsia^5^ has highlighted this gap in knowledge and the consequent need for more large surveys to obtain reliable estimates and inform policy. The data from the China Labor and Delivery Survey (CLDS) conducted throughout China^15^ offers a unique resource, which can be analyzed by inverse probability weighting (IPW)^15,16^ to provide country-level estimates that enable the comparison of a middle-income country with a high-income country. The present study uses 2 population-based data resources, the Swedish Medical Birth Register (MBR) and the CLDS to compare the prevalence, risk factors, and pregnancy outcomes for mild preeclampsia and severe preeclampsia between the 2 countries.

## Methods

### Study Populations

The MBR records maternal, pregnancy and neonatal information for nearly all deliveries in Sweden. During the study period (2007-2012), the register included deliveries at 22 weeks gestation or more.^17^ From these deliveries, there were 555 446 records available for analysis after excluding mothers with chronic hypertension.

In the CLDS, conducted from March 1, 2015 to December 31, 2016,^15^ information on more than 80 000 deliveries was extracted by trained research nurses in 89 hospitals from 24 of 34 provinces and autonomous regions in China. In this study, we included a total of 79 243 deliveries of at least 22 weeks gestation to women without chronic hypertension.

Ethical approvals were obtained from the Stockholm Regional Ethics Committee and the Xinhua Hospital Ethics Committee Affiliated to Shanghai Jiaotong University School of Medicine. The deidentified linkage of the Swedish national register data did not require informed consent. Because only anonymous clinical information was collected in the China Labor and Delivery Survey, no individual informed consent was obtained.^15^ We followed the Strengthening the Reporting of Observational Studies in Epidemiology (STROBE) reporting guideline.

### Identification of Hypertensive Disorders in Pregnancy

In Sweden, preeclampsia was diagnosed by physicians according to the guidelines of the Swedish Society of Obstetrics and Gynaecology^18^: mild preeclampsia was identified as gestational hypertension with proteinuria (≥300 mg/24 hours); if any of the 8 additional conditions in eTable 1 in the Supplement occurred, the preeclampsia was classified as severe. The diagnosis was recorded as *International Statistical Classification of Diseases and Related Health Problems, Tenth Revision *(*ICD-10*) codes in the MBR: gestational hypertension (*ICD-10* code O13), mild preeclampsia (*ICD-10* code O14.0), severe preeclampsia (*ICD-10* codes O14.1 and O14.9), hemolysis, elevated liver enzymes, low platelet count (HELLP) syndrome (*ICD-10* code O14.2), or eclampsia (*ICD-10* code O15). The diagnosis criteria in the Chinese data set followed the guidelines of the Chinese Society of Obstetrics and Gynecology^19^ with almost identical definitions as Sweden (eTable 1 in the Supplement), except for some additional conditions for classifying the diagnosis as severe. We classified pregnancies from both countries into 3 groups: no gestational hypertension (as the reference), mild preeclampsia, and severe preeclampsia. HELLP syndrome and eclampsia were included in the severe preeclampsia group. If more than 1 hypertensive disorder was registered, the pregnancy was classified according to the most severe diagnosis.

### Identification of Adverse Pregnancy Outcomes

Live births before 37 completed weeks were classified as preterm, according to the World Health Organization (WHO) definition.^20^ All pregnancies were classified according to whether they resulted in a liveborn or stillborn neonate. A 5-minute Apgar score of less than 7 was defined as low.^21^ Birth weights less than 500 g or greater than 7000 g were considered unknown.

### Definition of Potential Risk Factors

Maternal body mass index (BMI; calculated as weight in kilograms divided by height in meters squared) was calculated at first prenatal care visit in Sweden and classified using the WHO definition^22^(underweight, <18.5; reference weight, 18.5-24.9; overweight, 25.0-29.9; obese, ≥30.0). In China, prepregnancy BMI was categorized using the Chinese cutoffs, which are slightly different than the WHO definition^23^ (underweight, <18.5; reference weight, 18.5-23.9; overweight, 24-27.9; obese, ≥28). Binary (yes or no) risk factors included advanced maternal age (≥35 years at delivery), parous or nulliparous, multiple gestation, history of diabetes, and gestational diabetes (Table 1).

**Table 1.** Overall Demographic Description of the Pregnant Women in the Swedish and Chinese Populations^a^

Characteristic	No. (%)		*P* value
	Sweden	China	
No.	555 446	79 243	
Age, y			
Mean (SD)	30.91 (5.25)	28.63 (4.61)	<.001
<35	445 615 (80.2)	70 242 (88.9)	<.001
≥35	109 831 (19.8)	9001 (11.1)	
Prepregnancy			
BMI, mean (SD)	24.62 (4.47)	21.87 (3.29)	<.001^b^
Underweight	12 685 (2.3)	7163 (11.6)	<.001^c^
Reference weight	321 441 (57.9)	38 051 (66.6)	
Overweight	133 416 (24.0)	9931 (17.2)	
Obesity	63 846 (11.5)	2693 (4.6)	
Missing	24 058 (4.3)	21 405 (27.0)^c^	
Parity			<.001
Nulliparous	244 417 (44.0)	44 250 (54.3)	
Parous	311 029 (56.0)	34 5915 (45.7)	
Multiple gestation			<.001
Yes	7578 (1.4)	1733 (1.8)	
No	547 868 (98.6)	77 510 (98.2)	
History of diabetes			<.001
Yes	3190 (0.6)	816 (0.8)	
No	552 256 (99.4)	77 996 (99.2)	
Gestational diabetes			<.001^b^
Yes	5692 (1.0)	7799 (9.8)	
No	549 754 (99.0)	70 349 (88.8)	
Missing^c^	NA	1095 (1.4)	
Cesarean delivery			<.001
Yes	95 091(17.1)	29 124 (34.5)	
No	460 355 (82.9)	49 781 (65.5)	
Hypertensive disorders in pregnancy			.002
Total	22 089 (3.98)	3383 (4.02)	
Gestational hypertension	6021 (1.1)	1580 (1.8)	
Preeclampsia			
Mild	10 138 (63.1)	575 (31.9)	
Severe	5222 (32.5)	1228 (68.1)	
Unspecified	708 (4.4)	NA	

Abbreviations: BMI, body mass index calculated as weight in kilograms divided by height in meters squared; NA, not applicable.

^a^ Values for China are presented as observed mean (SD) or observed/unweighted No. (weighted %).

^b^ χ^2^ test of nonmissing categories.

^c^ The percentage of missing vales was 1.4% for gestational diabetes and 27% for BMI, all other variables were at least 99.5% complete.

### Statistical Analysis

Descriptive comparisons between Sweden and China used χ^2^ tests for categorical variables and student *t* tests for continuous variables. The Chinese multilevel survey recorded pregnancies within hospitals and provinces. Because population and hospital statistics were available, the overall epidemiology of preeclampsia in China can be represented by the application of IPW to this complex survey data.^24^ The proportion of participating hospitals in each province, representing the probability that a hospital was represented in the survey, was calculated separately for secondary and tertiary hospitals, using the numbers of hospitals in each province in 2016.^25^ Because, each hospital contributed all their delivery records for a specified period, these were assumed to be a representative proportion of the annual number of births in the hospital in 2016.^15^ The inverse of the product of these 2 proportions and probabilities provides the appropriate weight for an IPW analysis to obtain population estimates.

Univariable and multivariable logistic regression models were used to estimate the crude and adjusted odds ratios (ORs) for the association between potential risk factors and preeclampsia, with weighted logistic models used for the Chinese data. For the univariable regression model, any variable statistically significant in either country was reported (Table 2). Because maternal age, maternal BMI, parity, multiple gestation, history of diabetes, and gestational diabetes have been previously reported and have been validated as clinical risk factors for preeclampsia,^26^ these factors were all adjusted for in the multivariable regression models, regardless of statistical significance. For the separate analysis of mild preeclampsia and severe preeclampsia, the adjusted ORs with 95% CIs are presented on forest plots.

**Table 2.** Crude OR (95% CI) From Logistic Regression Analysis

	Preeclampsia, crude OR (95% CI)			
	Mild		Severe	
Variable	Sweden	China^**a**^	Sweden	China^**a**^
Maternal age at delivery ≥35	1.07 (1.02-1.13)	2.06 (1.22-3.48)	1.14 (1.06-1.22)	2.04 (1.59-2.63)
BMI^b^				
Underweight	0.76 (0.63-0.91)	0.41 (0.18-0.92)	0.78 (0.62-0.99)	0.66 (0.45-0.95)
Overweight	1.71 (1.63-1.80)	3.14 (1.98-4.99)	1.35 (1.26-1.45)	2.29 (1.59-3.30)
Obese	3.23 (3.07-3.40)	4.86 (3.64-6.51)	2.10 (1.94-2.27)	4.46 (3.27-6.07)
Nulliparous	2.49 (2.39-2.59)	1.40 (0.88-2.22)	3.31 (3.10-3.53)	1.20 (0.92-1.55)
Multiple gestation	4.42 (4.04-4.85)	2.57 (1.49-4.46)	5.76 (5.12-6.47)	6.21 (4.60-8.38)
History of diabetes	4.62 (4.04-5.29)	1.31 (0.59-2.90)	5.36 (4.47-6.43)	3.15 (1.37-7.27)
Gestational diabetes	2.97 (2.63-3.35)	1.23 (0.80-1.91)	2.29 (1.88-2.80)	2.13 (1.58-2.87)

Abbreviations: BMI, body mass index calculated as weight in kilograms divided by height in meters squared; OR, odds ratio.

^a^ Results for China are from weighted logistic regression.

^b^ Reference group: normal BMI.

The rates of preterm birth, low birth weight, and low 5-min Apgar score for pregnancies with mild and severe preeclampsia are illustrated using bar charts, and the Swedish and Chinese rates compared using (weighted) χ^2^ tests. Stillbirth rates are presented and tested for all women with preeclampsia (whether mild or severe) because all preeclamptic pregnancies resulting in stillbirth are coded as severe by the Chinese guidelines.^19^ Data management and analysis were conducted using SAS statistical analysis software, version 9.4 (SAS Institute, Inc), and figures were prepared using R statistical software, version 3.4.1 (R Project for Statistical Computing). A 2-sided *P* < .05 was considered statistically significant.

## Results

### Descriptive Results

**Figure 1.**
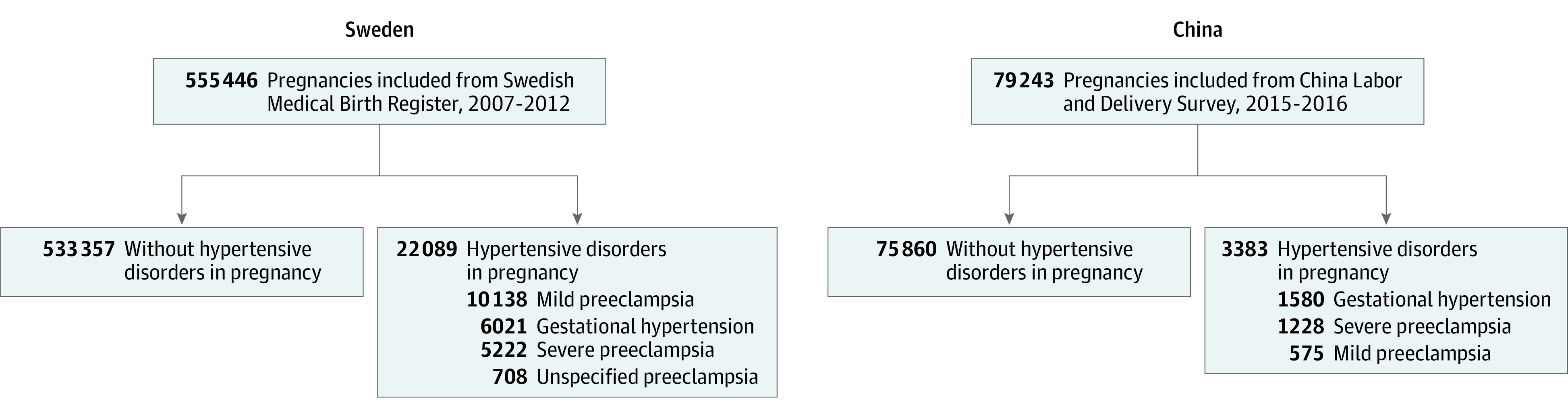
Flowchart of the Study Population in Sweden and Surveyed Sample in China

A total of 555 446 and 79 243 pregnancies were extracted from the Swedish Medical Birth Register and the China Labor and Delivery survey, respectively, with 22 089 (3.98%) and 3383 (4.27%) having a diagnosis of gestational hypertension (Figure 1). Of our sample, 76 315 (95) Chinese mothers were Han. The Swedish data recorded only maternal country of birth and not ethnic background (which may be mixed), and therefore the ethnic makeup of the sample could not be assessed. The mean (SD) maternal age was higher in mothers from Sweden than mothers from China (30.9 [5.6] years vs 28.6 [4.6] years). The mean (SD) BMI was also higher in mothers from Sweden than mothers from China (24.6[4.5] vs 21.9 [3.3]). The obesity rate in Sweden was more than double the rate in China (63 846 [11.5%] vs 2693 [4.6%]) (Table 1). There were 244 417 first deliveries (44.0%) in Sweden and 44 250 (54.3%) in China.

The prevalence of chronic diabetes and gestational diabetes were both significantly higher in China than in Sweden (chronic diabetes: 816 [0.8%] vs 3190 [0.6], *P* < .001; gestational diabetes: 7799 [8.4%] vs 5692 [1.0%], *P* < .001. The multiple gestation rate was similar in the 2 countries (7578 [1.4%] in Sweden and 1733 [1.8%] in China), and women who were nulliparous accounted for approximately half the deliveries in both countries (244 417 [44.0%] in Sweden vs 44 250 [54.3%] in China). More cesarean deliveries were performed in China than in Sweden (29 124 [34.5%] vs 95 091 [17.1%]).

The overall prevalence of preeclampsia was similar in Sweden and China, 16 068 (2.9%) and 1803 (2.3%), respectively. The overall prevalence of hypertensive disorders was almost identical in Sweden and China (22 089 [3.98%] and 3383 [4.02%]). However, there was a significant difference in the proportions of mild and severe preeclampsia. Of all preeclampsia cases, mild preeclampsia was more prevalent in Sweden than in China (10 138 [63.1%] vs 575 [31.9%]), while severe preeclampsia was less prevalent in Sweden (5222 [32.5%] vs 1228 [68.1%] in China; *P* < .001).

### Risk Factors for Preeclampsia

Table 2 presents crude ORs for the association of risk factors with preeclampsia. Maternal age of 35 years or older was significantly associated with both mild and severe preeclampsia in Sweden and China (mild: OR, 1.07; 95% CI, 1.02-1.13 and OR, 2.06; 95% CI, 1.22-3.48; severe: OR, 1.14; 95% CI, 1.06-1.22 and OR, 2.04; 95% CI, 1.59-2.63). Multiple gestation was significantly associated with both mild and severe preeclampsia in Sweden and China (mild: OR, 4.42; 95% CI, 4.04-4.85 and OR, 2.57; 95% CI, 1.49-4.46; severe: OR, 5.76; 95% CI, 5.12-6.47 and 95% CI, 6.21; OR, 4.60-8.38). Having overweight was significantly associated with both mild and severe preeclampsia in Sweden and China (mild: OR, 1.71; 95% CI, 1.63-1.80 and OR, 3.14; 95% CI, 1.98-4.99; severe: OR, 1.35; 95% CI, 1.26-1.45 and OR, 2.29; 95% CI, 1.59-3.30). Having obesity was significantly associated with both mild and severe preeclampsia in Sweden and China (mild: OR, 3.23; 95% CI, 3.07-3.40 and OR, 4.86; 95% CI, 3.64-6.51; severe: OR, 2.10; 95% CI, 1.94-2.27 and OR, 4.46; 95% CI, 3.27-6.07). In Sweden, a significantly higher risk of mild and severe preeclampsia was associated with nulliparity, history of diabetes, and gestational diabetes (nulliparity: OR, 2.49; 95% CI, 2.39-2.59 and OR, 3.31; 95% CI, 3.10-3.53; history of diabetes: OR, 4.62; 95% CI, 4.04-5.29 and OR, 5.36; 95% CI, 4.47-6.43; and gestational diabetes: OR, 2.97; 95% CI, 2.63-3.35 and OR, 2.29; 95% CI, 1.88-2.80). In China there was no significant association with parity, and diabetes was only associated with the risk of severe preeclampsia (history of diabetes: OR, 3.15; 95% CI, 1.37-7.27 and gestational diabetes: OR, 2.13; 95% CI, 1.58-2.87).

**Figure 2.**
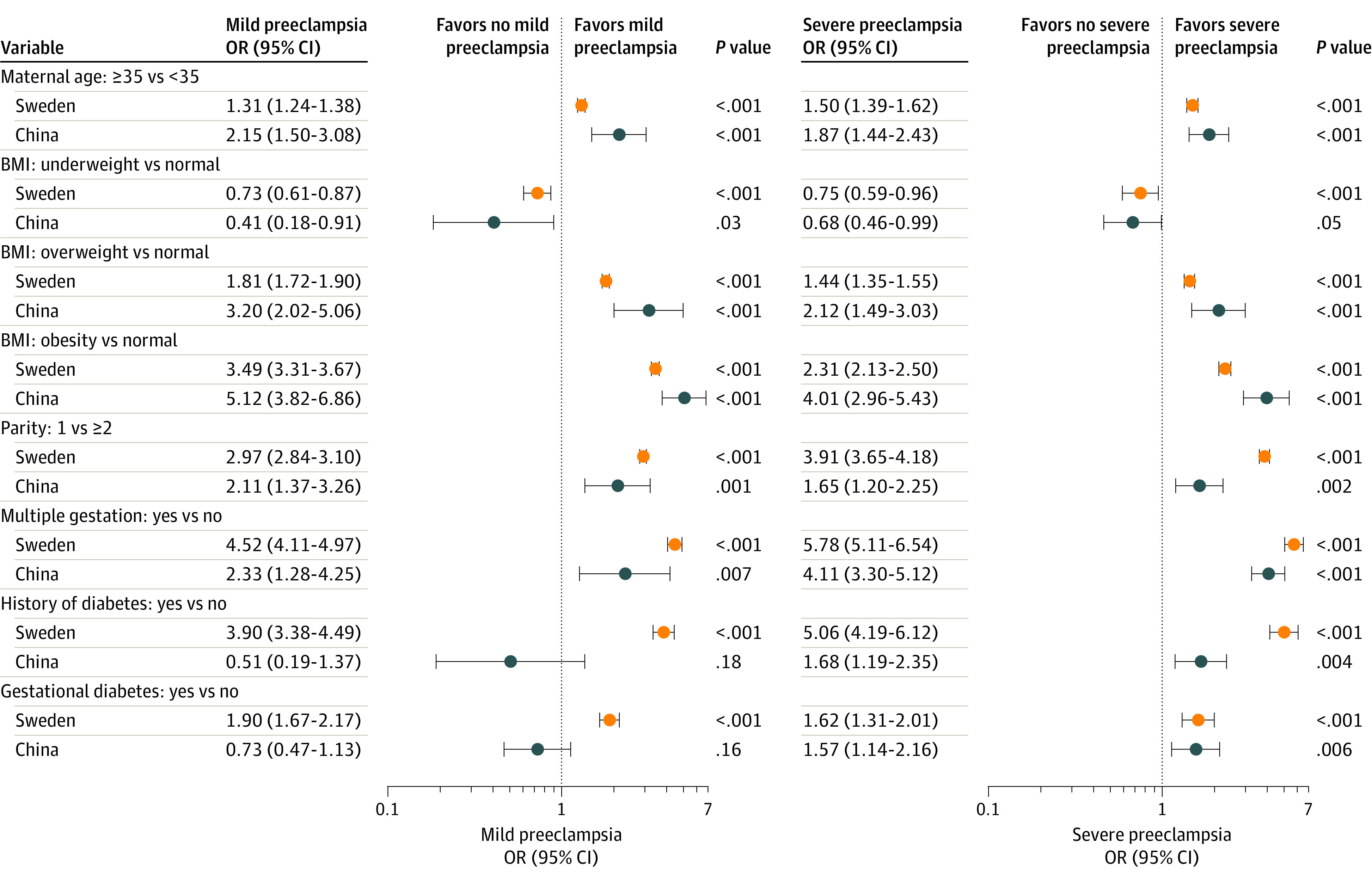
Adjusted Odds Ratios for Risk Factors Associated With Mild Preeclampsia and Severe Preeclampsia From Multivariable Logistic Regression for Sweden and Weighted Logistic Regression for China

The adjusted ORs (aORs) and 95% CIs are presented in Figure 2 (eTable 2 in the Supplement). Maternal age of 35 years or older had a weaker association with mild and severe preeclampsia in Sweden than in China (mild: aOR, 1.31; 95% CI 1.24-1.38 vs aOR, 2.15; 95% CI, 1.50-3.08; severe: aOR, 1.50; 95% CI, 1.39-1.62 vs aOR, 1.87; 95% CI, 1.44-2.43).

Women in China who had overweight or obesity had a 3-fold risk and 5-fold risk of mild preeclampsia (OR, 3.20; 95% CI, 2.02-5.06 and OR, 5.12; 95% CI, 3.82-6.86) and a 2-fold and 4-fold risk of severe preeclampsia (OR, 2.12; 95% CI, 1.49-3.03 and OR, 4.01; 95% CI; 2.96-5.43) compared with women with reference weight. All of the associations with overweight and obesity were stronger in women in China than women in Sweden.

Women in Sweden who were nulliparous had a 3- and 4-fold higher risk of mild and severe preeclampsia (OR, 2.97; 95% CI, 2.84-3.10 and OR, 3.91; 95% CI, 3.65-4.18), which were higher than the relative risks for women in China. Multiple gestation was associated with at least a 4-fold increased risk of severe preeclampsia in Sweden and China (OR, 5.78; 95% CI, 5.11-6.54 and OR 4.11, 95% CI, 3.30-5.12) and with mild preeclampsia in Sweden (OR 4.52, 95% CI, 4.11-4.97).

A history of diabetes was associated with mild preeclampsia and severe preeclampsia in Sweden (OR, 3.90; 95% CI, 3.38-4.49 and OR, 5.06; 95% CI, 4.19-6.12), while in China there was no association with mild preeclampsia and a weak association with severe preeclampsia (OR, 0.51; 95% CI, 0.19-1.37 and OR, 1.68; 95% CI, 1.19-2.35). In Sweden, associations with gestational diabetes were weaker than with having a history of diabetes but still significant, while in China the associations were of similar magnitude to those for chronic diabetes and only significant for severe preeclampsia (eTable 2 in the Supplement).

### Pregnancy Outcomes

The neonatal outcomes for singleton pregnancies were significantly different between the 2 countries and are presented in eTable 3 in the Supplement. China had higher overall rates of stillbirth than Sweden (846 [1.1%] vs 1753 [0.3%]), preterm birth (6157 [6.7%] vs 24 870 [4.5%]) and low birth weight (4105 [4.6%] vs 15 710 [2.9%]), while the prevalence of low 5-minute Apgar score was somewhat lower (801 [1.0%] vs 6889 [1.3%]). Mean gestational age at delivery and birth weight were significantly higher in Sweden (median [interquartile range, IQR], 40 [39.0-41.0] wk vs 39.0 [38.0-40.0] wk, and median [IQR], 3555 [3220-3890] g vs 3300 [3000-3600] g). All comparisons were significant at *P* < .001.

**Figure 3.**
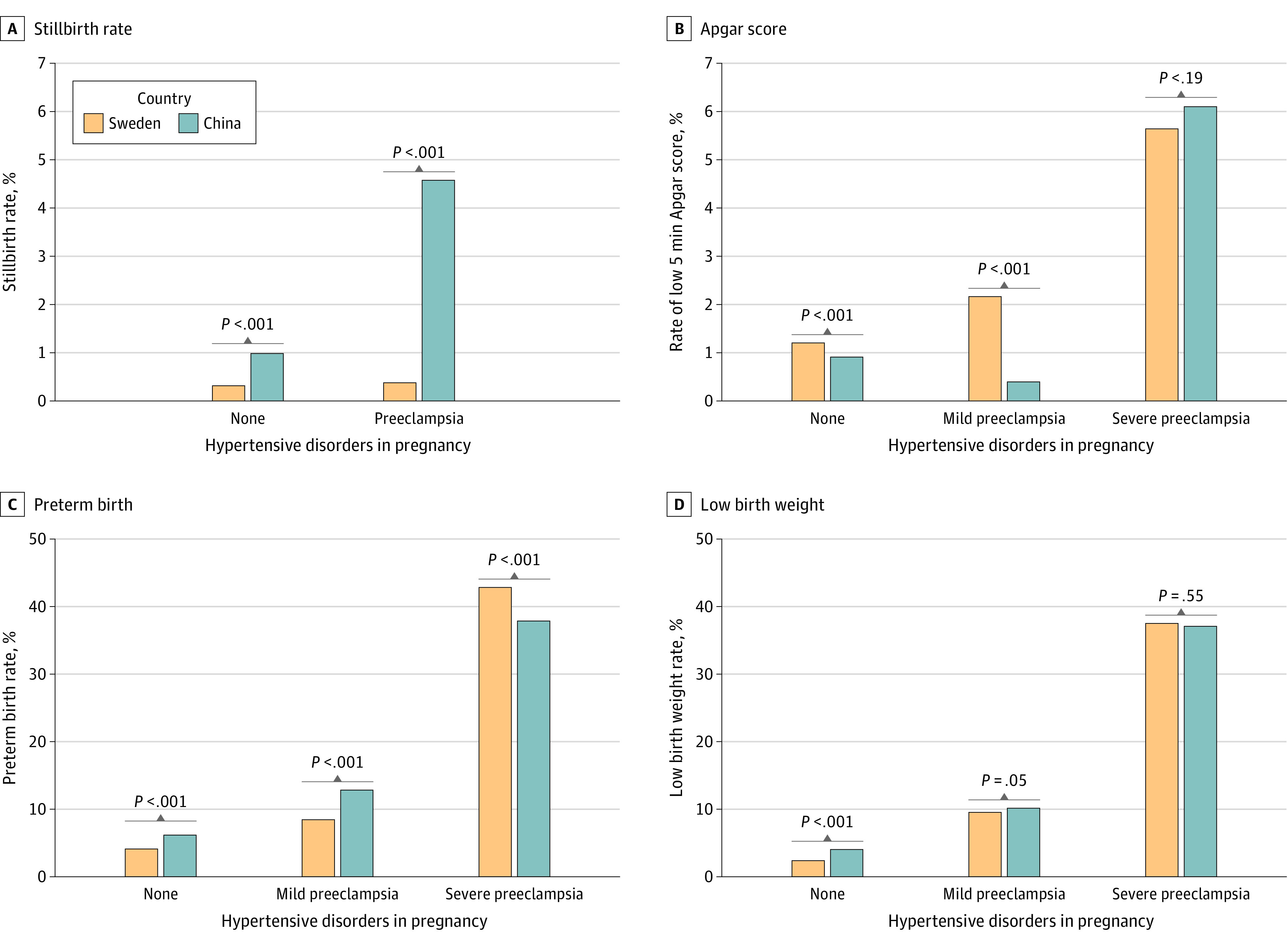
Comparison of Adverse Outcomes of Singleton Pregnancies Associated With Preeclampsia in Sweden and China Pregnancies with no gestational hypertension are included as reference.

Figure 3 presents a comparison of singleton pregnancy outcomes in Sweden and China, for pregnancies with no gestational hypertension, mild preeclampsia, and severe preeclampsia (eTable 4 in the Supplement). Multiple pregnancies are not presented because of insufficient data. The stillbirth rate for singleton pregnancies complicated by preeclampsia was 66 of 1652 (4.6%) in the Chinese survey data, 10-fold higher than in Sweden (60/14 499; 0.4%). A low 5-minute Apgar score was associated with both mild and severe preeclampsia in Sweden but only with severe preeclampsia in China, where it was of much larger magnitude than Sweden.

Both Sweden and China had an increasing rate of low birth weight and preterm birth rate across the 3 categories of exposure (no gestational hypertension, mild preeclampsia, severe preeclampsia). The proportions of low birth weights were similar in Sweden and China, with 916 (9.5%) and 50 (10.1%) in the mild preeclampsia group and 1821 (37.5%) and 453 (37.1%) in the severe group. The preterm rate in Sweden was significantly lower than China for mild preeclampsia, 812 (8.5%) vs 77 (12.8%), and significantly higher for severe preeclampsia, 2,094 (42.8%) vs 515 (37.9), with *P* < .001 for both comparisons.

## Discussion

Our results highlight several differences between Sweden and China regarding the presentation and risk factors of preeclampsia and the outcomes of affected pregnancies. The overall prevalence of gestational hypertensive disorders was approximately 4% in both countries, but the ratio between mild and severe preeclampsia was reversed: two-thirds of the cases were mild in Sweden, while two-thirds of the cases were severe in China. We found notable differences in the associations with known risk factors in the 2 populations: maternal age and obesity had a milder association in Sweden, while in China, obesity was associated with a 4- to 5- fold increased risk; nulliparity was a risk factor in both populations, but with a much stronger association in Sweden. The stillbirth rate for singleton pregnancies in the overall surveyed population was 1.1% in China, which was more than 3 times as high as in Sweden (0.3%). For pregnancies complicated by preeclampsia, the risk of stillbirth in women in China was 4.6% while it was only 0.4% in Sweden.

Multiple gestation, which was a strong risk factor in both countries, is a known risk factor for preeclampsia, with a 2-fold increased risk reported in United States populations of Black women and White women.^27^ In our study, the larger odds ratios of 4 to 5 in Sweden are consistent with a 4-fold increase in Norway.^28^

Ethnicity has previously been suggested as a risk factor for preeclampsia in a study within China^29^ and may explain some of the differences with Sweden. However, China has a significant Han majority (approximately 92% of the population), while Sweden is less homogenous, with approximately 80% of the population born in Sweden, and many of those may have a mixed ethnic background. There are many aspects of ethnicity, such as lifestyle, diet, and the seeking and provision of health care, that are likely to be more strongly associated with preeclampsia risk than the racial or genetic background. For example, in our study, obesity was a much stronger risk factor for women in China. Increased risks of 2- to 3-fold have been reported for non-Hispanic White or non-Hispanic Black women with obesity.^30,31^ Furthermore, a meta-analysis^32^of White populations from North America or Europe reported a relative risk of 4.14 (95% CI, 3.61-4.75) for risk of preeclampsia in women with BMI of more than 35 compared with a BMI of less than 25.

Several metabolic perturbations have been suggested as responsible for the association between obesity and preeclampsia, such as elevated leptin, proinflammatory status, or dysfunction of the nitric oxide synthase system.^33^ Our findings show that the risk of preeclampsia for women in China rises more rapidly with BMI than for Swedish women. The recording of prepegnancy BMI in China and the BMI in Sweden at the first prenatal visit is unlikely to explain much of this difference because we would not expect much change in this period.^34^ One hypothesis is that metabolic perturbations may appear earlier with increased BMI in Asian women, who have higher levels of visceral adiposity,^35^ which is associated with cardiovascular and metabolic disease.^36^

The high stillbirth rate in the overall Chinese population is consistent with the literature^37^ and more than 3 times as high as the stillbirth rate in Sweden. Worldwide, stillbirth rates are difficult to assess, as this indicator was not part of the WHO Millennium Development Goals and therefore not recorded in several countries, remaining an invisible aspect of adverse pregnancy outcome.^38^ Instead, the neonatal mortality rate is used for between-country comparisons, and huge disparities are observed.^38^ Our data, which was from 1 high-income and 1 middle-income country, reflects only a small part of this variation. However, it is compelling to observe that the risk of stillbirth was higher in women in China with preeclampsia (4.6%) than in the general population (1.1%), while in Sweden the rate only increased from 0.3% to 0.4%. Increased adverse outcomes, including stillbirths, have been reported for low- and middle-income countries,^39^ and the magnitude of the difference we observed between Sweden and China is more likely because of cultural and health care factors^11^ than genetic variation. We considered a number of potential explanations for the differences, including the protocols for antenatal care, association of prematurity with stillbirth risk, and duration of in-hospital care. The management of maternal health care in China or the care-seeking behavior of pregnant women might result in preeclampsia not being identified until symptoms have become more severe. The typical schedule for routine prenatal follow-up in both countries includes 4 visits up to week 32: following this, China has 2 visits in weeks 33 to 36 and 37 to 41, while Sweden has visits every second week until delivery.^40^ Thus, women in China have less surveillance in late pregnancy, when preeclampsia onset is more likely. Differences in health care are also evident from the high overall cesarean delivery rate (34.5%) in China (double that of Sweden’s 17.1%) which is consistent with other studies.^41,42^

The management of preeclampsia before gestational week 37 is challenging in low- and middle-income countries.^39^ To accommodate the WHO definition of stillbirth,^43^ we stratified by gestational age at delivery (<28 weeks, 28-31 weeks, 32-36 weeks, and ≥37 weeks). Among extremely preterm deliveries (<28 weeks), the stillbirth rate was 16.5% in Sweden and 80% in China, and among very preterm (28-31 weeks), it was 1.3% and 28%. The stillbirth rate was low in both countries for full-term deliveries, but there was still a 4-fold difference (0.11% in Sweden vs 0.48% in China). We hypothesized that women in China may seek medical help later, with a result that intrauterine death has already occurred when they arrive at the hospital. However, 33 of 45 extremely and very preterm deliveries (73.3%) were more than 24 hours after hospitalization, suggesting that the fetus was alive when the woman reached maternity care. Thus, the high stillbirth rates are more likely to be due to aspects of expectant care^44^ combined with unexpected complications. The contribution of maternal factors and surveillance protocols is an important area for further investigation.

Regarding genetic factors, it has long been known that there is familial clustering of preeclampsia,^7^ and more recently, discoveries of genetic markers associated with plausible biological angiogenic pathways have been made.^12,45^ Asian and women in China have been underrepresented in genetic studies of preeclampsia. Our work provides a comparison of an Asian and a European country that are not genetically homogenous populations, but our results nonetheless suggest that genetic studies focused on ethnic Chinese women could provide important insights. Additional population-based epidemiological studies, such as comparing women in China with their migrant counterparts, could also help to unravel the genetic and environmental contributions to preeclampsia risk.

A strength of this study is the availability of representative population data in a high-income and middle-income country, enabling a between-country comparison and a contribution to the global depiction of preeclampsia. The MBR captures almost every birth in Sweden using *ICD* codes that have been reported to have high validity for identifying preeclampsia (positive predictive value of 92.6%) in the Swedish context.^46^

The participating provinces in the CLDS represent 92% of the population (and 89% of the births) during the years of the survey. Thus, our weighted estimates represent most of the Chinese population. By taking account of the numbers and sizes of secondary and tertiary hospitals in the weighted analysis, we mitigated the potential bias from referral patterns or participation rates in the survey.

### Limitations

This study has limitations. Although almost identical diagnosis criteria were used for mild preeclampsia in the 2 countries, the Chinese definition included some additional criteria for the classification of the condition as severe (eTable 1 in the Supplement). Most of these additional criteria are too rare to explain the higher prevalence of severe preeclampsia, but fetal growth restriction is relatively common, and its strong association with preeclampsia^47^ may have resulted in some diagnostic or misclassification bias.

The numbers of study mothers from the 2 countries were unbalanced, with the Chinese survey sample being approximately 14% of the Swedish study population. Despite the smaller sample size, the Chinese data provided estimates with good precision and had sufficient statistical power to identify important differences with Sweden.

Although the Swedish prevalence is for a time (2007-2012) when preeclampsia rates were shown to be stable by our group (eFigure in the Supplement) and others,^48^ we cannot exclude the possibility of confounding by unmeasured factors during this 6-year period. The Chinese prevalence is estimated during 2015 to 2016, so any comparison with Sweden during the same calendar year(s) would require the assumption that the Swedish rates do not change substantially for a further 3 to 4 years. Interestingly, the 2015 to 2016 prevalence in China was remarkably close to that of Sweden during a 6-year period. Given this similarity, an interesting area for further investigation is whether the proportion of severe disease and associated adverse outcomes in China have reduced in recent years.

A major limitation of our study is the lack of information about differences in the management of preeclampsia. There were also no details about treatment available for either country. A further limitation of the data from both countries is that the date of preeclampsia onset or diagnosis was not available, so that we were unable to separate early and late-onset preeclampsia.

While our study is nationally representative, we do not have the necessary information to estimate the association with ethnic or genetic factors. Although 95% of the Chinese survey sample were Han women (similar to 92% nationally), we cannot assume our results apply to this ethnic group without having the population and hospital statistics stratified by ethnic group for the IPW estimation. The Swedish data records maternal country of birth, not ethnic background (which may be mixed), so these data can only be interpreted as representative of a country and not of one or more ethnic groups.

## Conclusions

In this study, important differences were found between women in Sweden and China in the overall rate of severe preeclampsia, the contributions of risk factors, and the fetal outcomes for affected pregnancies. While the differences in the association with parity and BMI are likely because of ethnic and social factors, the higher prevalence of severe disease and rate of adverse pregnancy outcomes in China may reflect differences in national protocols for antenatal care and the management of pregnancies that are complicated by preeclampsia. An underlying genetic association or potential difference in pathophysiology between Chinese and European women cannot be ruled out. Variation in the contributions of risk factors across populations could be important for global efforts to identify early serum markers of preeclampsia and also have consequences for the development of complex prediction models for pregnancies at risk of preeclampsia.^49,50^ Our work suggests that comparative studies of preeclampsia need to consider diagnostic differences, social and ethnic factors, and various aspects of health care protocols. While this study has highlighted the potential importance of national health care protocols in comparing different countries, differences in health care may also be important in the interpretation of findings from within-country studies of multiethnic populations.

## Notes

Supplement. **eTable 1.** Summary of Chinese Hypertensive Disorders in Pregnancy Guidelines (2015 Edition) and Translated Guidelines From the Swedish Society of Obstetrics and Gynecology **eTable 2. **Adjusted Odds Ratios From Multivariable Logistic Regression Model **eTable 3. **Neonatal Outcomes of Singleton Pregnancies in Sweden and China **eTable 4. **Comparison of Pregnancy Outcomes Among Singleton Pregnancies in Sweden and China **eFigure.** Annual Prevalence of Hypertensive Disorders in Pregnancy in Sweden from 2002 to 2012

## References

[1] Kuklina EV, Ayala C, Callaghan WM. Hypertensive disorders and severe obstetric morbidity in the United States. Obstet Gynecol. 2009;113(6):1299-1306. doi:10.1097/AOG.0b013e3181a45b25 19461426

[2] Tranquilli AL, Brown MA, Zeeman GG, Dekker G, Sibai BM; Statements from the International Society for the Study of Hypertension in Pregnancy (ISSHP). The definition of severe and early-onset preeclampsia. Pregnancy Hypertens. 2013;3(1):44-47. doi:10.1016/j.preghy.2012.11.001 26105740

[3] Poon LC, Shennan A, Hyett JA, . Erratum to “the International Federation of Gynecology and Obstetrics (FIGO) initiative on pre-eclampsia: a pragmatic guide for first-trimester screening and prevention” [Int J Gynecol Obstet 145 Suppl. 1 (2019) 1-33]. Int J Gynaecol Obstet. 2019;146(3):390-391. doi:10.1002/ijgo.12892 31378938

[4] Brown MA, Magee LA, Kenny LC, ; International Society for the Study of Hypertension in Pregnancy (ISSHP). The hypertensive disorders of pregnancy: ISSHP classification, diagnosis and management recommendations for international practice. Pregnancy Hypertension. 2018;13:291-310. doi:10.1016/j.preghy.2018.05.004 29803330

[5] Abalos E, Cuesta C, Grosso AL, Chou D, Say L. Global and regional estimates of preeclampsia and eclampsia: a systematic review. Eur J Obstet Gynecol Reprod Biol. 2013;170(1):1-7. doi:10.1016/j.ejogrb.2013.05.005 23746796

[6] Galaviz-Hernandez C, Sosa-Macias M, Teran E, Garcia-Ortiz JE, Lazalde-Ramos BP. Paternal determinants in preeclampsia. Front Physiol. 2019;9:1870. doi:10.3389/fphys.2018.01870 30666213 PMC6330890

[7] Cnattingius S, Reilly M, Pawitan Y, Lichtenstein P. Maternal and fetal genetic factors account for most of familial aggregation of preeclampsia: a population-based Swedish cohort study. Am J Med Genet A. 2004;130A(4):365-371. doi:10.1002/ajmg.a.30257 15384082

[8] Nakagawa K, Lim E, Harvey S, Miyamura J, Juarez DT. Racial/ethnic disparities in the association between preeclampsia risk factors and preeclampsia among women residing in Hawaii. Matern Child Health J. 2016;20(9):1814-1824. doi:10.1007/s10995-016-1984-2 27000850 PMC5007163

[9] Marić I, Mayo JA, Druzin ML, . Maternal height and risk of preeclampsia among race/ethnic groups. Am J Perinatol. 2019;36(8):864-871. doi:10.1055/s-0038-1675205 30396225

[10] Johnson JD, Louis JM. Does race or ethnicity play a role in the origin, pathophysiology, and outcomes of preeclampsia? an expert review of the literature. Am J Obstet Gynecol. 2020;S0002-9378(20)30769-9. doi:10.1016/j.ajog.2020.07.038 32717255

[11] Gong J, Savitz DA, Stein CR, Engel SM. Maternal ethnicity and pre-eclampsia in New York City, 1995-2003. Paediatr Perinat Epidemiol. 2012;26(1):45-52. doi:10.1111/j.1365-3016.2011.01222.x 22150707 PMC4169658

[12] Srinivas SK, Morrison AC, Andrela CM, Elovitz MA. Allelic variations in angiogenic pathway genes are associated with preeclampsia. Am J Obstet Gynecol. 2010;202(5):445.e1-445.e11. doi:10.1016/j.ajog.2010.01.040 20223440

[13] Kaartokallio T, Klemetti MM, Timonen A, . Microsatellite polymorphism in the heme oxygenase-1 promoter is associated with nonsevere and late-onset preeclampsia. Hypertension. 2014;64(1):172-177. doi:10.1161/HYPERTENSIONAHA.114.03337 24799610

[14] Lv X, Li X, Dai X, . Investigation *heme oxygenase-1* polymorphism with the pathogenesis of preeclampsia. Clin Exp Hypertens. 2020;42(2):167-170. doi:10.1080/10641963.2019.1601202 30978117

[15] Chen C, Zhang JW, Xia HW, . Preterm birth in China between 2015 and 2016. Am J Public Health. 2019;109(11):1597-1604. doi:10.2105/AJPH.2019.305287 31536409 PMC6775901

[16] Korn EL, Graubard BI. Epidemiologic studies utilizing surveys: accounting for the sampling design. Am J Public Health. 1991;81(9):1166-1173. doi:10.2105/AJPH.81.9.1166 1951829 PMC1405642

[17] Stephansson O, Petersson K, Björk C, Conner P, Wikström AK. The Swedish pregnancy register—for quality of care improvement and research. Acta Obstet Gynecol Scand. 2018;97(4):466-476. doi:10.1111/aogs.13266 29172245 PMC5873375

[18] Swedish Association for Obstetrics and Gynecology Work and Reference Group for Perinatology. Preeclampsia. Accessed March 9, 2021. https://www.sfog.se/natupplaga/ARG72_komplett_LRbf4f5598-2309-4013-8dea-3cbb534708ee.pdf

[19] Hypertension Disorders of Pregnancy Group, Chinese Society of Obstetrics and Gynecology, Chinese Medical Association. Diagnosis and treatment guideline for hypertension disorders of pregnancy. Chin J Obstet Gynecol 2015; 50:721-728. doi:10.3760/cma.j.issn.0529-567X.2015.10.001.5

[20] World Health Organization. Preterm birth. Published February 19, 2018. Accessed March 9, 2021. https://www.who.int/news-room/fact-sheets/detail/preterm-birth

[21] Siddiqui A, Cuttini M, Wood R, ; Euro-Peristat Scientific Committee. Can the Apgar Score be used for international comparisons of newborn health? Paediatr Perinat Epidemiol. 2017;31(4):338-345. doi:10.1111/ppe.12368 28621463

[22] World Health Organization. WHO: recommended definitions, terminology and format for statistical tables related to the perinatal period and use of a new certificate for cause of perinatal deaths. modifications recommended by FIGO as amended October 14, 1976. Acta Obstet Gynecol Scand. 1977;56(3):247-253. 560099

[23] He W, Li Q, Yang M, . Lower BMI cutoffs to define overweight and obesity in China. Obesity (Silver Spring). 2015;23(3):684-691. doi:10.1002/oby.20995 25645003

[24] Mansournia MA, Altman DG. Inverse probability weighting. BMJ. 2016;352:i189. doi:10.1136/bmj.i189 26773001

[25] National Bureau of Statistics of China. China statistical yearbook 2016. Accessed March 1, 2021. http://www.stats.gov.cn/tjsj/ndsj/2016/indexch.htm

[26] Paré E, Parry S, McElrath TF, Pucci D, Newton A, Lim KH. Clinical risk factors for preeclampsia in the 21st century. Obstet Gynecol. 2014;124(4):763-770. doi:10.1097/AOG.0000000000000451 25198274

[27] Sibai BM, Hauth J, Caritis S, ; National Institute of Child Health and Human Development Network of Maternal-Fetal Medicine Units. Hypertensive disorders in twin versus singleton gestations. Am J Obstet Gynecol. 2000;182(4):938-942. doi:10.1016/S0002-9378(00)70350-4 10764477

[28] Sole KB, Staff AC, Laine K. The association of maternal country of birth and education with hypertensive disorders of pregnancy: a population-based study of 960 516 deliveries in Norway. Acta Obstet Gynecol Scand. 2018;97(10):1237-1247. doi:10.1111/aogs.13393 29873810

[29] Xiao J, Shen F, Xue Q, . Is ethnicity a risk factor for developing preeclampsia? an analysis of the prevalence of preeclampsia in China. J Hum Hypertens. 2014;28(11):694-698. doi:10.1038/jhh.2013.148 24430700

[30] Bodnar LM, Ness RB, Markovic N, Roberts JM. The risk of preeclampsia rises with increasing prepregnancy body mass index. Ann Epidemiol. 2005;15(7):475-482. doi:10.1016/j.annepidem.2004.12.008 16029839

[31] Catov JM, Ness RB, Kip KE, Olsen J. Risk of early or severe pre-eclampsia related to pre-existing conditions. Int J Epidemiol. 2007;36(2):412-419. doi:10.1093/ije/dyl271 17255351

[32] Kalliala I, Markozannes G, Gunter MJ, Obesity and gynaecological and obstetric conditions: umbrella review of the literature. BMJ. 2017;359:j4511. doi:10.1136/bmj.j4511 PMC5656976 29074629

[33] Spradley FT. Metabolic abnormalities and obesitys impact on the risk for developing preeclampsia. Am J Physiol Regul Integr Comp Physiol. 2017;312(1):R5-R12. doi:10.1152/ajpregu.00440.2016 27903516 PMC5283940

[34] Johansson K, Hutcheon JA, Bodnar LM, Cnattingius S, Stephansson O. Pregnancy weight gain by gestational age and stillbirth: a population-based cohort study. BJOG. 2018;125(8):973-981. doi:10.1111/1471-0528.15034 29160923 PMC6032856

[35] Lim U, Ernst T, Buchthal SD, . Asian women have greater abdominal and visceral adiposity than Caucasian women with similar body mass index. Nutr Diabetes. 2011;1(5):e6. doi:10.1038/nutd.2011.2 PMC3302135 23449381

[36] Karlsson T, Rask-Andersen M, Pan G, . Contribution of genetics to visceral adiposity and its relation to cardiovascular and metabolic disease. Nat Med. 2019;25(9):1390-1395. doi:10.1038/s41591-019-0563-7 31501611

[37] Xiong T, Mu Y, Liang J, . Hypertensive disorders in pregnancy and stillbirth rates: a facility-based study in China. Bull World Health Organ. 2018;96(8):531-539. doi:10.2471/BLT.18.208447 30104793 PMC6083384

[38] Lawn JE, Gravett MG, Nunes TM, Rubens CE, Stanton C; GAPPS Review Group. Global report on preterm birth and stillbirth (1 of 7): definitions, description of the burden and opportunities to improve data. BMC Pregnancy Childbirth. 2010;10:S1. doi:10.1186/1471-2393-10-S1-S1 PMC2841772 20233382

[39] Beardmore-Gray A, Vousden N, Charantimath U, Planned early delivery versus expectant management to reduce adverse pregnancy outcomes in pre-eclampsia in a low- and middle-income setting: study protocol for a randomised controlled trial (CRADLE-4 Trial). BMC Pregnancy Childbirth. 2020;21(1):960. doi:10.1186/s13063-020-04888-w PMC7684962 33228794

[40] Chinese Society of Obstetrics and Gynecology, Chinese Medical Association. Guideline for prenatal care and antenatal care (edition 1). Chin J Obstet Gynecol. 2011;46:150-153. doi:10.3760/cma.j.issn.0529-567x.2018.01.003

[41] Li HT, Luo S, Trasande L, . Geographic variations and temporal trends in cesarean delivery rates in China, 2008-2014. JAMA. 2017;317(1):69-76. doi:10.1001/jama.2016.18663 28030701

[42] Liang J, Mu Y, Li X, . Relaxation of the one child policy and trends in caesarean section rates and birth outcomes in China between 2012 and 2016: observational study of nearly seven million health facility births. BMJ. 2018;360:k817. doi:10.1136/bmj.k817 PMC5836714 29506980

[43] World Health Organization. Maternal, newborn, child and adolescent health. Accessed July 22, 2020. https://www.who.int/maternal_child_adolescent/epidemiology/stillbirth/en/

[44] Churchill D, Duley L, Thornton JG, Moussa M, Ali HS, Walker KF. Interventionist versus expectant care for severe pre-eclampsia between 24 and 34 weeks' gestation. Cochrane Database Syst Rev. 2018;10(10):CD003106. doi:10.1002/14651858.CD003106.pub3 PMC6517196 30289565

[45] Schmella MJ, Roberts JM, Conley YP, . Endoglin pathway genetic variation in preeclampsia: a validation study in Norwegian and Latina cohorts. Pregnancy Hypertens. 2018;12:144-149. doi:10.1016/j.preghy.2017.10.005 29580923 PMC5995147

[46] Ludvigsson JF, Andersson E, Ekbom A, External review and validation of the Swedish national inpatient register. BMC Public Health. 2011;11:450. doi:10.1186/1471-2458-11-450 PMC3142234 21658213

[47] Odegård RA, Vatten LJ, Nilsen ST, Salvesen KA, Austgulen R. Preeclampsia and fetal growth. Obstet Gynecol. 2000;96(6):950-955. 11084184

[48] Roberts CL, Ford JB, Algert CS, Population-based trends in pregnancy hypertension and pre-eclampsia: an international comparative study. BMJ Open. 2011;1(1):e000101. doi:10.1136/bmjopen-2011-000101 PMC3191437 22021762

[49] De Kat AC, Hirst J, Woodward M, Kennedy S, Peters SA. Prediction models for preeclampsia: a systematic review. Pregnancy Hypertens. 2019;16:48-66. doi:10.1016/j.preghy.2019.03.005 31056160

[50] Chaemsaithong P, Sahota DS, Poon LC. First trimester preeclampsia screening and prediction. Am J Obstet Gynecol. 2020;S0002-9378(20)30741-9. doi:10.1016/j.ajog.2020.07.020 32682859

