# Supplementary material for: Preeclampsia Prevalence, Risk Factors, and Pregnancy Outcomes in Sweden and China
Source: JAMA Netw Open. 2021 May 10;4(5):e218401. doi: 10.1001/jamanetworkopen.2021.8401 (PMC8111481; doi:10.1001/jamanetworkopen.2021.8401)
Supplement: Supplement. — eTable 1. Summary of Chinese Hypertensive Disorders in Pregnancy Guidelines (2015 Edition) and Translated Guidelines From the Swedish Society of Obstetrics and Gynecology eTable 2. Adjusted Odds Ratios From Multivariable Logistic Regression Model eTable 3. Neonatal Outcomes of Singleton Pregnancies in Sweden and China eTable 4. Comparison of Pregnancy Outcomes Among Singleton Pregnancies in Sweden and China eFigure. Annual Prevalence of Hypertensive Disorders in Pregnancy in Sweden from 2002 to 2012 [file jamanetwopen-e218401-s001.pdf]

Supplemental Online Content

Yang Y, Le Ray I, Zhu J, Zhang J, Hua J, Reilly M. Preeclampsia prevalence, risk factors, and pregnancy outcomes in Sweden and China. *JAMA Netw Open.* 2021;4(5):e218401.
doi:10.1001/jamanetworkopen.2021.8401

eTable 1. Summary of Chinese Hypertensive Disorders in Pregnancy Guidelines (2015 Edition) and Translated Guidelines From the Swedish Society of Obstetrics and Gynecology
eTable 2. Adjusted Odds Ratios From Multivariable Logistic Regression Model
eTable 3. Neonatal Outcomes of Singleton Pregnancies in Sweden and China
eTable 4. Comparison of Pregnancy Outcomes Among Singleton Pregnancies in Sweden and China
eFigure. Annual Prevalence of Hypertensive Disorders in Pregnancy in Sweden from 2002 to 2012

This supplemental material has been provided by the authors to give readers additional information about their work.

© 2021 Yang Y et al. *JAMA Network Open.*

eTable 1. Summary of Chinese Hypertensive Disorders in Pregnancy Guidelines (2015 Edition)[1] and Translated Guidelines From the Swedish Society of Obstetrics and Gynecology[2]

	China	Sweden
Gestational hypertension	Systolic BP≥140 mmHg and (or) diastolic BP ≥90 mmHg after 20 weeks gestation in a woman with a previously normal blood pressure	Blood pressure >=140 mm Hg systolic and 90 mm Hg diastolic, measured on two occasions at least 4 hours apart, after 20 weeks pregnancy.
Preeclampsia	Gestational hypertension and proteinuria (>=0.3g/24h or protein / creatinine ratio>=0.3 or dipstick reading of 1+ (used only if other quantitative methods not available) OR (in the absence of proteinuria) new-onset hypertension with onset of any of (i) impaired organ (ii) abnormality of blood, digestive or nervous system (iii) Placenta-fetal involvement	Gestational hypertension and proteinuria<br>(≥300 mg / 24 hours)
Severe preeclampsia	preeclampsia with any of the following:<br>(1) Continuously increasing hypertension: systolic blood pressure ≥ 160 mmHg and (or) diastolic blood pressure ≥ 110 mmHg<br>(2) Cerebral or visual symptoms: continuous headache or visual disturbance<br>(3) Impaired liver: continuous upper abdominal pain and hepatic subcapsular hematoma or liver rupture<br>(4) Abnormally elevated blood concentrations of liver enzymes (ALT or AST)<br>(5) Impaired renal function: proteinuria ≥ 2.0g/24h, oliguria (urine < 400 ml/24h, or <17ml/h)<br>(6) Hypoproteinemia with ascites, pleural effusion or pericardial effusion<br>(7) Thrombocytopenia: platelet count <100*10^9/L; microvascular hemolysis (anemia, jaundice or elevated LDH)<br>(8) Heart failure<br>(9) Pulmonary edema<br>(10) Fetal growth restriction, oligohydramnios, fetal death or placental abruption	Preeclampsia with any of the following:<br>(1) systolic blood pressure ≥160 mm Hg (on two occasions with at least 4 hours interval unless on antihypertensive treatment)<br>(2) diastolic blood pressure ≥110 mm Hg (on two occasions with at least 4 hours interval unless on antihypertensive),<br>(3) new onset cerebral symptoms or visual changes<br>(4) severe pain in the epigastric area or under the right arc without other etiology<br>(5) affected liver enzymes (twice the upper normal value)<br>(6) oliguria (<500 ml / day),<br>(7) pulmonary edema or cyanosis,<br>(8) thrombocytopenia (<100 x10^9 / L),
HELLP	(1) Intravascular hemolysis: the presence of broken red blood cells and spherical red blood cells in peripheral blood smear; Bilirubin ≥ 20.5 μmol/L (1.2 mg/dl); Hemoglobin slightly decreased; the level of LDH increased.<br>(2) Increased liver enzyme levels: ALT≥40 U/L or AST≥70 U/L.<br>(3) Decrease in platelet count: platelet count< 100×10^9/L.	Hemolysis determined by haptoglobin or LD, platelets <100 x10^9 / L, and ASAT or ALAT ≥ 1.2μkat/L (equal to 70 U/L).
Eclampsia	The presence of general convulsions in a woman with preeclampsia, which cannot be explained by other causes occur.	General convulsions during pregnancy, childbirth or the first few weeks after delivery in patients with preeclampsia that cannot be explained by other causes (for example, epilepsy).

© 2021 Yang Y et al. *JAMA Network Open.*

Source: 1. Hypertension disorders of pregnancy Group, Chinese Society of Obstetrics and Gynecology, Chinese Medical Association. Diagnosis and treatment Guideline for hypertension disorders of pregnancy (2015), Chin J Obstet Gynecol, 2015; 50(10):721-728.
2. https://www.sfog.se/natupplaga/ARG72_komplett_LRbf4f5598-2309-4013-8dea-3cbb534708ee.pdf

© 2021 Yang Y et al. *JAMA Network Open.*

eTable 2. Adjusted Odds Ratios From Multivariable Logistic Regression Model[a]

	Mild Preeclampsia		Severe Preeclampsia	
**Variable**	**Sweden**	**China (weighted)**	**Sweden**	**China (weighted)**
Maternal age at delivery >=35	1.31 (1.24-1.38)	2.15 (1.50-3.08)	1.50 (1.39-1.62)	1.87 (1.44-2.43)
BMI*				
Underweight	0.73 (0.61-0.87)	0.41 (0.18-0.91)	0.75 (0.59-0.96)	0.68 (0.46-0.99)
Overweight	1.81 (1.72-1.90)	3.20 (2.02-5.06)	1.44 (1.35-1.55)	2.12 (1.49-3.03)
Obese	3.49 (3.31-3.67)	5.12 (3.82-6.86)	2.31 (2.13-2.50)	4.01 (2.96-5.43)
Nulliparous	2.97 (2.84-3.10)	2.11 (1.37-3.26)	3.91 (3.65-4.18)	1.65 (1.20-2.25)
Multiple gestation	4.52 (4.11-4.97)	2.33 (1.28-4.25)	5.78 (5.11-6.54)	4.11 (3.30-5.12)
History of diabetes	3.90 (3.38-4.49)	0.51 (0.19-1.37)	5.06 (4.19-6.12)	1.68 (1.19-2.35)
Gestational diabetes Mellitus	1.90 (1.67-2.17)	0.73 (0.47-1.13)	1.62 (1.31-2.011	1.57 (1.14-2.16)

*Reference group: normal BMI.

[a]Results for China are from weighted logistic regression.

© 2021 Yang Y et al. *JAMA Network Open.*

eTable 3. Neonatal Outcomes of Singleton Pregnancies in Sweden and China[a,b]

	Sweden	China	P-value
N	(547219)	77,512	
Gestational age at delivery (weeks)	40.0 (39.0-41.0)	39.0 (38.0-40.0)	<0.001
Preterm	24870 (4.5)	6,157 (6.7)	<0.001
Birth weight (g)	3555 (3220-3890)	3300 (3000-3600)	<0.001
Low birth weight	15710 (2.9)	4,105 (4.6)	<0.001
Low 5-minute Apgar score	6889(1.3)	801 (1.0)	<0.001
Stillbirth	1753 (0.3)	846 (1.1)	<0.001

[a]Values for China are presented as weighted median (quartiles) or observed/unweighted n (weighted %).
[b]Median (quartiles) are reported for continuous variables and n(%) for categorical variables.

© 2021 Yang Y et al. *JAMA Network Open*.

eTable 4. Comparison of pregnancy outcomes among singleton pregnancies in Sweden and China[a].

	Sweden	China	P	Sweden	China	P
N	9611	535		4888	1,117	
Gestational age	39.0 (38.0-40.0)	38.0 (37.0-39.0)	<0.001	37.0 (35.0-39.0)	37.0 (34.0-38.0)	0.006
Preterm	812(8.5)	77 (12.8)	<0.001	2,094 (42.8)	515 (37.9)	<0.001
Birth weight	3400 (2965-3825)	3300 (2900-3650)	<0.001	2785 (2125-3345)	2700 (1970-3230)	<0.001
Low birthweight*	916 (9.5)	50 (10.1)	0.05	1821(37.5)	453 (37.1)	0.55
Low 5-minute Apgar score*	207 (2.2)	1 (0.4)	<0.001	273 (5.6)	58 (6.1)	0.19
Stillbirth	27 (0.3)	0 (0)	-	33 (0.7)	66 (6.9)	<0.001

[a]Values for China are presented as weighted median (quartiles) or observed/unweighted n (weighted %).
[b]Median (quartiles) are reported for continuous variables and No. (%) for categorical variables.
[c]Low birth weight available in Swedish data for 9605 and 4856 of mild and severe respectively, and low Apgar score for 9569 and 4840.

© 2021 Yang Y et al. *JAMA Network Open.*

eFigure. Annual Prevalence of Hypertensive Disorders in Pregnancy in Sweden from 2002 to 2012

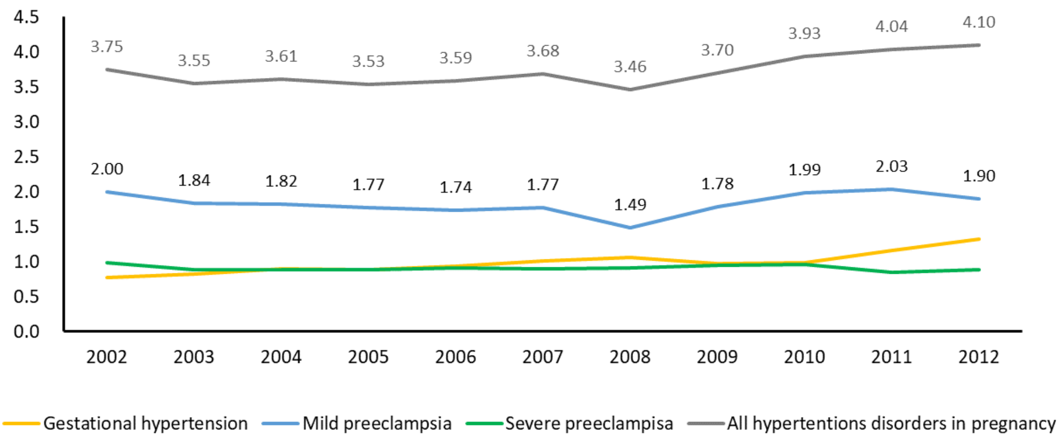

© 2021 Yang Y et al. *JAMA Network Open*.
